# The Election of Direct Representatives

**Published:** 1896-08-01

**Authors:** 


### The Hospital

An Institutional Journal of

The Medical Sciences and Hospital Administration, with

AN EXTRA NURSING SUPPLEMENT.

Vol. XX.—No. 514.] [Aug. 1, 1896.

### The Election of Direct Representatives.

If representative government be politically good, the medical profession should insist upon having a reasonable share of it. We assume for the moment that it is good. According to the present constitution of the General Medical Council, the profession can hardly be said to enjoy even a modicum of representative government. For in a Council consisting of a total of thirty members, only five of whom are elected by the unfettered choice of the profession, it is difficult to imagine that much influence can be exercised on the whole body by the voting power of the five. But for this very reason it is the more imperative that the direct representatives, when chosen, be men of intelligence, courage, and determination, in order that they may make up for their lack of voting power by political intelligence and fighting capacity. When we say "fighting capacity," we do not mean "blustering capacity." Between "fighting" and "blustering" there is all the difference in the world.

The candidates who are most influentially "backed" in the present contest are Dr. Glover; Dr. Woodcock, of Manchester; and Dr. Lovell Drage, of Hatfield; and since in the medical profession "backing" is the single *sine quâ non* of acceptance, it is highly probable that these three will be elected. Of Dr. Glover something is known, and that something is favourable to his claims. He has for ten years represented his brother general practitioners with dignity and intelligence, and has probably influenced the other members of the Council to regard with a more equal feeling their professional brethren who follow general practice. If this be so, Dr. Glover deserves well of the rank and file of the profession, and ought undoubtedly to be re-elected.

Dr. Woodcock is not widely known to the profession. But his Lancashire brethren who have put him forward as their representative may be trusted to know a man of capacity when they see him. He has selected a decidedly fighting platform. In his address at the meeting of July 16th he expressed the conviction that the "General Medical Council had proved itself to be one of the greatest hindrances to the advancement of the profession with which the profession had to deal. The one great obstacle to the amendment of the Medical Acts was the General Mtdical Council. He held that not only did the constitution of the Council require altering, but also its temper, its disposition, and its feeling with regard to the aspirations of the profession." These are bold words, and it is felt throughout the profession that they contain at least some truth. Experience shows, however, that it is not always the boldest speaker on an electioneering platform who makes the most strenuous fighter when the period of actual warfare arrives. Will Dr. Woodcock, if elected, enter upon the prodigious task of reforming the constitution of the Medical Council, and of improving its temper and disposition? Above all, will he, if he once enters upon so unique a task, bring to bear upon the accomplishment of it tact, energy, unyielding courage, and a perseverance which knows no limits? If he does, he may in time hope to effect something definite; and at any rate he will prove himself to be a living man among semi-fossilised conventionalities, a vital force in the midst of a sapless forest of platitudinarians.

Of Dr. Lovell Drage not much can, as yet, be said. His brother, Mr. Geoffrey Drage, M.P., has shown a considerable measure of political capacity; and Dr. Lovell Drage has proved that he does not lack energy and courage of a certain kind. Whether he will show as bold a front in the presence of the venerable notabilities of the Medical Council as he has shown upon the more popular platforms of a somewhat crude medical reform remains to be seen. He is a young man of dash and spirit, who, having taken an Oxford degree, is necessarily endowed with a certain tincture of liberal culture, and has been accustomed to measure himself by the standard of men who have played and will play conspicuous parts in the greater world of general politics and affairs. And these are distinct advantages.

On the whole the three men whom we have named present three distinct types of the modern general practitioner, and it will be felt that they are fairly representative of general practice as it is. Looking about among the other candidates who have issued addresses, we cannot see that any of them are more likely to give real expression to what are called the "aspirations of the profession" than these three. Some of the other candidates are, we hold, decidedly unsuitable, and it is very undesirable that they should be elected. It is a pity, we venture to believe, that general practitioners prefer to be represented by general practitioners alone. Far more hopeful would it be for their "aspirations" if they would look out for strong, intelligent, and courageous representatives, without regard to their professional specialties. They would thus have a much wider range of choice, and would certainly secure for themselves a more practically effective representation. But since general practitioners, and they only, find favour with the bulk of the profession, it is probable that the three we have named will constitute as good a selection as could, without a very great deal of difficulty, have been made.

